# Microbial Primer: what is the stringent response and how does it allow bacteria to survive stress?

**DOI:** 10.1099/mic.0.001483

**Published:** 2024-07-30

**Authors:** Lucy Urwin, Orestis Savva, Rebecca M. Corrigan

**Affiliations:** 1 The Florey Institute of Infection, School of Biosciences, University of Sheffield, Sheffield, S10 2TN, UK; 2 The School of Medicine, University College Dublin, Belfield, Dublin 4, Ireland

**Keywords:** ppGpp, stress, stringent response

## Abstract

The stringent response is a conserved bacterial stress response that allows bacteria to alter their activity and survive under nutrient-limiting conditions. Activation of the stringent response is characterized by the production of intracellular signalling molecules, collectively termed (p)ppGpp, which interact with multiple targets inside bacterial cells. Together, these interactions induce a slow growth phenotype to aid bacterial survival by altering the transcriptomic profile of the cell, inhibiting ribosome biosynthesis and targeting enzymes involved in other key metabolic processes.

## How do bacteria deal with stress?

Bacteria are ubiquitous in our environment and display remarkable adaptability to changing conditions. This is largely attributable to the presence of complex signalling networks that allow bacteria to sense and respond to environmental stresses. These stresses include, but are not limited to, extremes of temperature or pH, DNA damage, oxidative stress, changes in osmotic pressure and nutrient starvation conditions[1]. Bacteria have evolved numerous mechanisms that allow them to cope with these stresses, and this protective toolbox includes both general stress responses that are induced non-specifically in response to sub-optimal growth conditions and adaptive stress responses that are associated with a specific environmental trigger.

General stress responses are governed by sigma (*σ*) factors that function to guide the core RNA polymerase (RNAP) to promoter sequences, facilitating the transcription of DNA into RNA. Different *σ* factors display different promoter sequence specificity and so are associated with the transcription of different subsets of genes. Over 100 different *σ* factors have been identified across bacterial species, but bacteria generally possess one housekeeping protein and multiple alternative *σ* factors. For example, *σ*^A^ (*rpoD*) is the major sigma factor utilized by *Escherichia coli* and is responsible for transcribing most genes under normal growth conditions, but this bacterium also possesses multiple alternative sigma factors, including *σ*^F^ (*rpoF*), *σ*^H^ (*rpoH*), *σ*^N^ (*rpoN*) and *σ*^S^ (*rpoS*). *σ*^S^ is considered the general stress response sigma factor and is produced in response to unfavourable conditions, including during the transition from the exponential to stationary growth phases. As *σ*^s^ accumulates, it replaces *σ*^A^ in the RNAP holoenzyme, and RNAP is redirected to initiate transcription at alternative promoter sequences.

In contrast, adaptive stress responses mediate a more tailored response to specific environmental cues. One well-characterized example of this is the SOS response, whereby the accumulation of single-stranded DNA (ssDNA) signals to the cell that DNA damage has occurred. This ssDNA forms a complex with RecA, which ultimately triggers the transcription of DNA repair genes, including low-fidelity DNA polymerases that are normally repressed. In this way, bacteria can survive severe DNA damage by sensing ssDNA and upregulating specific genes to counteract the stress. Similarly, the accumulation of unfolded proteins can trigger the production of chaperone proteins as part of the heat shock response, and high intracellular proton concentrations can induce changes in bacterial cell membrane permeability as part of the acid stress response, demonstrating the breadth of these specialized stress responses. However, for the purposes of this primer, we will focus solely on the specific bacterial stress response triggered by nutrient-limiting conditions – the stringent response.

## What is the stringent response?

The stringent response is an intracellular signalling network controlled by a family of phosphorylated nucleotides termed (p)ppGpp. Activation of the stringent response refers to a surge in intracellular (p)ppGpp levels, and this typically occurs when bacteria are exposed to nutrient-limiting, or so-called ‘stringent’, conditions that threaten bacterial survival. (p)ppGpp is a collective term used to describe two nucleotides that function jointly as the alarmones (i.e. intracellular signalling molecules) of the stringent response: guanosine tetraphosphate (ppGpp) and guanosine pentaphosphate (pppGpp). These alarmones were first discovered by Cashel and Gallant over 50 years ago and are sometimes referred to as the ‘magic spot’ alarmones because they were first observed as unknown spots on a thin layer chromatogram used to separate nucleotides from starved *E. coli* cell lysates [2].

The enzymes responsible for synthesizing (p)ppGpp are referred to as RSH (RelA-SpoT homologue) enzymes, and they take their name from the two (p)ppGpp synthetases first discovered in *E. coli* (RelA and SpoT) [3]. This family of enzymes is ubiquitous in bacteria, with the absence of *rsh* genes only reported in bacterial species that inhabit extremely stable microenvironments and/or exist as obligate intracellular parasites (e.g. *Chlamydia* species). However, there are several key differences in the structure and function of RSH enzymes between different classes of bacteria (Table 1), as well as multiple interspecies differences, and so for the remainder of this primer, *E. coli* and *Staphylococcus aureus* will be described as well-studied representatives of the Gammaproteobacteria and Bacilli classes, respectively.

**Table 1.** Key features of the stringent response in two model species of bacteria, *E. coli* and *S. aureus*

Model organism	*E. coli*	*S. aureus*
**Bacterial phylum and class**	Pseudomonadota, Gammaproteobacteria (*γ*)	Firmicutes, Bacilli
**RSH enzymes**	**Long RSH:** most *β*- and *γ*-proteobacteria possess two long RSH enzymes believed to have arisen from a gene duplication event. In *E. coli*, these are RelA and SpoT. Other Proteobacteria typically possess one long RSH enzyme**Short RSH (SAS/SAH):** rare in Proteobacteria, not present in *E. coli*	**Long RSH:** generally possess one long RSH enzyme, which is referred to as Rel in *S. aureus* (previously RSH)**Short RSH (SAS/SAH):** commonly found in Firmicutes. *S. aureus* possesses two small alarmone synthetases, RelQ and RelP
**Examples of conditions that induce (p)ppGpp synthesis**	**RelA:** amino acid or nitrogen starvation**SpoT:** fatty acid starvation	**Rel:** amino acid starvation**RelQ/P:** cell wall stress**RelP:** ethanol, alkaline shock
**Effects on cell activity**	**Transcription:** (p)ppGpp directly regulates transcription by binding RNAP**Translation:** inhibition of ribosome-associated proteins including biogenesis factor GTPases, initiation factor 2 (IF2), elongation factors (EF-Tu, EF-G), release factors (RF1-3) and ribosome recycling factor (RRF)**DNA replication:** DNA replication is inhibited post-translationally by (p)ppGpp-mediated inhibition of the DNA primase, DnaG. (p)ppGpp also inhibits chromosomal replication initiation from *oriC* via an unknown mechanism believed to involve initiator protein DnaA. Destabilization of RNAP complexes by (p)ppGpp may also reduce transcriptional activity near *oriC*, altering negative supercoiling and reducing replication	**Transcription:** (p)ppGpp indirectly regulates transcription by reducing cellular GTP levels. GTP acts as an initiating nucleotide during transcription and is an essential cofactor for the master transcriptional repressor, CodY. The ATP:GTP ratio is influenced by decreasing GTP, thereby altering the activity of promoters that use ATP as an initiating nucleotide**Translation:** as for *E. coli***DNA replication:** DNA replication is inhibited through binding to DnaG, as for *E. coli*

**Fig. 1.**
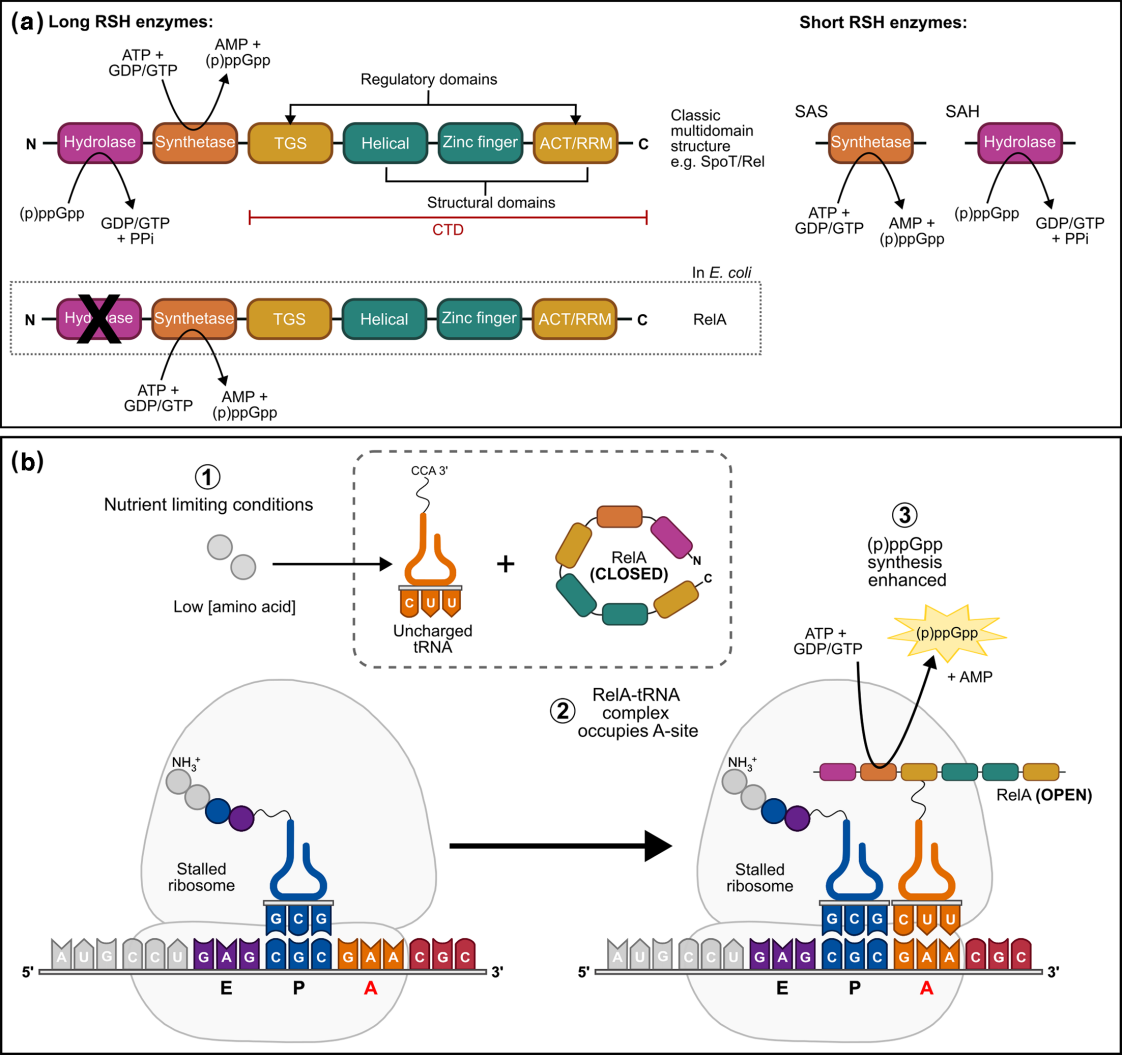
Basic structure and regulation of RSH enzymes. (**a**) Long RSH enzymes possess a multi-domain structure. Catalytic domains responsible for hydrolyzing and synthesizing (p)ppGpp are located at the N-terminus. Regulatory domains, TGS (Thr-tRNA synthase, GTPase, SpoT) and ACT (aspartokinase, chorismate mutase, TyrR), constitute the C-terminal domain (CTD) and are separated by two structural domains, one entirely helical domain and a ZFD composed of three conserved cysteine residues. The ACT domain is sometimes referred to as the RNA recognition motif (RRM) because it can bind RNA. Short RSH enzymes are monodomain proteins that either synthesize (small alarmone synthetase) or hydrolyse (small alarmone hydrolase) (p)ppGpp. (**b**) Ribosome-dependent regulation of the *E. coli* RelA occurs under starvation conditions. When amino acid concentrations are limiting, deacylated tRNA molecules accumulate inside the cell and ribosomes stall as elongation of the nascent polypeptide chain is disrupted. RelA complexes with uncharged tRNA in the ribosome A site in an ‘open’ conformation that is associated with enhanced (p)ppGpp synthetase activity. The RelA CTD plays an essential role in facilitating this interaction, with the TGS domain interacting with the CCA 3′ end of tRNA and specific interactions between ZFD-ACT and rRNA stabilizing the RelA–tRNA–ribosome complex.

RSH enzymes can be divided into two distinct categories, long and short RSH enzymes, based on the number of domains they possess (Fig. 1a). Long RSH enzymes are multi-domain structures with bifunctional enzymatic activity. At the N-terminus are the hydrolase domain (HD) and synthetase domain (SD). Synthesis occurs via the transfer of a pyrophosphate group from ATP to either GDP or GTP, generating ppGpp or pppGpp and a molecule of AMP. In this way, (p)ppGpp levels are directly tied to intracellular nucleotide concentrations, and following activation of the stringent response, intracellular pools of GDP/GTP are rapidly depleted. Conversely, the HD catalyses hydrolysis by removing a pyrophosphate group from (p)ppGpp to produce GDP or GTP. In addition to the HD/SD, long RSH enzymes also contain two structural domains [helical domain and zinc finger domain (ZFD)], as well as two regulatory domains (TGS and ACT) (Fig. 1a). Regulatory domains play an important role in regulating the activity of long RSH enzymes by mediating protein or ligand interactions and driving changes in RSH protein conformation. Of the two *E. coli* RSH enzymes, only SpoT displays bifunctional activity, as the HD of RelA is degenerate.

In contrast, short RSH enzymes are monodomain proteins that possess either (p)ppGpp synthetase or hydrolase activity. These enzymes are referred to as small alarmone synthetases (SAS) and small alarmone hydrolases (SAH), respectively. Short RSH enzymes are uncommon in Proteobacteria but are abundant among Firmicutes and bacteria belonging to this phylum typically possess two SAS enzymes, RelQ and RelP [4]. SAH proteins have not been identified in *S. aureus,* but whole-genome sequencing has identified candidate SAH genes in other bacterial species.

## How is the stringent response activated?

Regulation of RSH enzymes is a complex field, and so for the purposes of this primer, only major principles are described. (p)ppGpp is always present at basal levels in the cell. Cellular (p)ppGpp levels are controlled by the transcriptional regulation of RSH expression and the opposing activities of (p)ppGpp SD and HD, whose catalytic activity can be modulated by interactions with other cellular proteins, single-stranded RNA (ssRNA) and even (p)ppGpp molecules themselves[5]. Uncontrolled accumulation of (p)ppGpp is toxic to bacterial cells, and mutants that lack hydrolase activity are non-viable if synthetases are still present. However, large surges in intracellular (p)ppGpp are observed in response to nutrient-limiting conditions. Nutritional cues typically alter the transcriptional regulation and/or activity of long RSH enzymes, with inducing conditions including, but not limited to, amino acid starvation, nitrogen starvation and fatty acid deprivation. Amino acid starvation is considered the principal activator of the stringent response and has conserved features across *E. coli* and *S. aureus*. As part of this pathway, bacteria sense low amino acid concentrations via the accumulation of uncharged tRNAs and this stimulates long RSH enzymes to increase (p)ppGpp production in a ribosome-dependent manner. This mechanism has been well studied in *E. coli* (Fig. 1b), where RelA can form a complex with uncharged tRNA molecules and occupy the empty A site of stalled ribosomes [6]. This causes conformational changes in the RelA protein that enhance (p)ppGpp synthesis and induce the stringent response. In contrast, the short RSH enzymes, which lack a C-terminal regulatory region, are typically regulated at the transcriptional level, with increased expression of SAS enzymes observed in response to cell wall stress, ethanol and alkaline conditions.

## How does the stringent response work?

Once produced, the stringent response alarmones can target a plethora of different pathways, including transcription, translation and DNA replication. The versatility of this signalling molecule is largely attributable to its phosphate groups, which confer significant conformational flexibility and allow (p)ppGpp to bind multiple targets within the cell [7]. These targets are utilized by bacteria to slow down non-essential processes and enter a so-called hibernation state as a means of energy conservation and survival. We discuss a number of the most well-characterized pathways below.

### Transcription

**Fig. 2.**
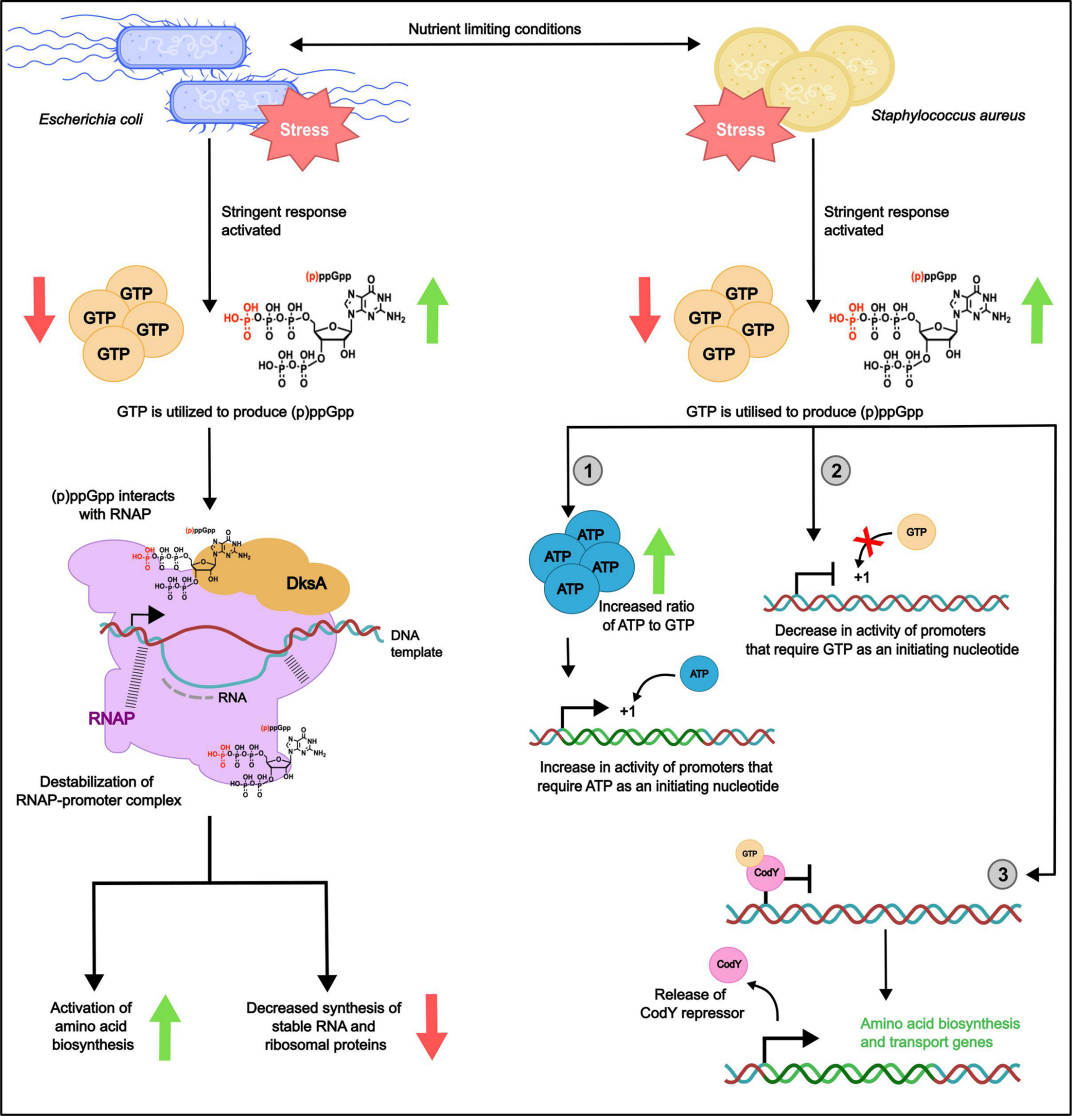
Effects of the stringent response on transcription. The stringent response alters transcription via differing mechanisms in Gammaproteobacteria (*E. coli*) and Bacilli (*S. aureus*). In *E. coli*, (p)ppGpp directly modifies transcription via its interaction with RNAP at two distinct binding sites. This destabilizes RNAP–promoter interactions with differing effects on different promoters, e.g. increased transcription of genes controlling amino acid synthesis and decreased transcription of stable RNA and ribosomal proteins. In *S. aureus*, (p)ppGpp indirectly regulates transcription via changes in intracellular nucleotide concentrations. There are three main mechanisms by which this occurs: (1) the drop in intracellular GTP that occurs following (p)ppGpp production increases the ATP:GTP ratio, thereby increasing transcription from promoters that utilize ATP as an initiating nucleotide; (2) conversely, there is a decrease in transcription from promoters that utilize GTP as an initiating nucleotide; (3) de-repression of the CodY regulon occurs as the master transcriptional repressor, CodY, cannot interact with its essential cofactor, GTP. Amino acid biosynthesis and transport genes are upregulated following CodY de-repression, contributing to bacterial survival in the absence of essential nutrients.

In *E. coli*, (p)ppGpp regulates transcription directly by binding to the RNAP (Fig. 2). The RNAP has two known (p)ppGpp binding sites that are conserved in Proteobacteria. The first is located between the *β*′ and *ω* subunits, while the second is located near the secondary channel of subunit *β*′, at a site formed upon binding of the transcription factor DksA. In the presence of (p)ppGpp, the tri complex of DksA, RNAP and (p)ppGpp results in the destabilization of RNAP–promoter complexes. This destabilization has different outcomes depending on the promoter, with activation of promoters controlling amino acid biosynthesis and inhibition of promoters responsible for production of stable ribosomal RNA and ribosomal proteins. Previous studies have shown that within minutes, over 750 RNA transcripts are affected as a direct result of the interaction between (p)ppGpp and the RNAP.

In *S. aureus* and other Firmicutes*,* the (p)ppGpp alarmones regulate transcription indirectly via changes in intracellular nucleotide concentrations (Fig. 2). Production of (p)ppGpp utilizes GTP as a substrate, thereby depleting cellular GTP, and in parallel, (p)ppGpp directly inhibits multiple enzymes required for GTP biosynthesis (e.g. PurF and Gmk) [8]. The associated reduction in GTP levels has both direct and indirect effects on transcription. Some promoters, including ribosomal RNA promoters, require GTP as an initiating nucleotide during transcription, and therefore, rRNA levels are decreased in response to (p)ppGpp production. The transcription of amino acid biosynthesis genes is indirectly controlled by the staphylococcal stringent response because GTP is an essential cofactor for the master transcriptional repressor CodY. In the presence of GTP, CodY represses the transcription of hundreds of genes, but following activation of the stringent response, cellular GTP levels drop and CodY de-repression occurs. Many of the genes controlled by CodY are involved in the biosynthesis and transport of amino acids, and therefore, CodY de-repression contributes to bacterial survival in the absence of essential nutrients. While the levels of GTP reduce upon induction of the stringent response, ATP concentrations remain stable, resulting in an increased ratio of ATP to GTP. This increases transcription from promoters that use ATP as an initiating nucleotide, many of which are involved in the production of branched-chain amino acids.

### Translation

The stringent response has negative effects on both ribosome maturation and function. The individual 50S and 30S subunits that form the 70S ribosome mature with the help of ribosome-associated GTPases (RA-GTPases), such as RbgA, RsgA, Era, Obg and HflX. These RA-GTPases are inhibited during the stringent response by the direct binding of (p)ppGpp, leading to a smaller pool of mature ribosomal subunits available for the formation of the 70S. In *E. coli*, (p)ppGpp can also induce the transcription of the genes *hpf* (ribosome hibernation promoting factor gene), *rmf* (ribosome modulation factor gene) and *raiA* (ribosome-associated inhibitor gene), which are essential for 70S dimerization into inactive 100S hibernating particles. Furthermore, in *S. aureus*, the RA-GTPase HflX is, in part, responsible for the dissociation of 100S ribosomes in a GTP-dependent manner, with its inhibition by (p)ppGpp leading to a greater accumulation of inactive 100S particles and fewer 70S active ribosomes.

Once the 70S is formed, it works continuously with interaction partners to translate mRNA to protein. (p)ppGpp can inhibit a number of these interaction partners, including initiation factor 2 (IF2), thereby preventing the formation of the 30S initiation complex, and the elongation factors EF-Tu and EF-G, which are essential for the delivery of amino acid–charged tRNAs and translocation of the peptide chain during protein synthesis, respectively. However, the inhibition of IF2 by (p)ppGpp depends on the mRNA bound to the pre-30S initiation complex, suggesting that essential proteins could still be translated even when the stringent response is active. When peptide synthesis is complete, (p)ppGpp can also inhibit the ribosome release factors RF1, RF2 and RF3, alongside the ribosomal recycling factor (RRF), all of which are required for ribosome recycling. Through all of the above, it is clear that (p)ppGpp controls nearly every facet of ribosome maturation and activity.

### DNA replication

The stringent response is also capable of altering chromosomal replication, and this occurs via different mechanisms in *E. coli* and *S. aureus*. Chromosomal replication is initiated at the origin of replication *oriC*. For replication to be initiated, the DnaA initiator protein must bind *oriC* in its ATP-bound form (DnaA-ATP), with various other enzymes also recruited for replication to proceed, including the DNA primase DnaG. The stringent response has been shown to inhibit chromosomal replication initiation in *E. coli,* but the exact mechanism by which this occurs is unknown. It has been suggested that reduced *dnaA* transcription during the stringent response could explain this phenomenon, coupled with activation of the Lon protease, which degrades DnaA-ADP. When DnaA-ADP is degraded, pathways within the cell convert DnaA-ATP to DnaA-ADP to restore balance between the two states, thus reducing DnaA-ATP levels even further. It has also been suggested that reduced transcriptional activation at *oriC* may play a role, with the activity of promoters located near *oriC* reduced, presumably due to the inhibition of RNAP. This inhibition of RNAP, and associated decrease in transcription, subsequently decreases negative supercoiling near the *oriC*, thus hindering the binding of DnaA to the origin. In contrast, the *S. aureus* stringent response has no direct effect on chromosomal replication initiation or RNAP activity. Instead, chromosomal replication is inhibited post-translationally by (p)ppGpp-mediated inhibition of DnaG, reducing the RNA primer synthesis needed for DNA replication, a mechanism that also occurs in *E. coli*.

## How does this help bacteria?

Overall, the stringent response and the (p)ppGpp alarmones produced have a variety of effects on different pathways within bacterial organisms. The aim of this stress response is to reduce the energy expenditure of the cell to a minimum, so as to ensure its survival under unfavourable conditions by allowing only the processes of utmost importance to proceed. This slow growth phenotype also has important implications in the context of human infections, as activation of the stringent response has been linked to reduced antibiotic efficacy and the development of chronic bacterial infections that are difficult to treat and recurrent in nature [9,10]. A clinical strain isolated from a patient chronically infected with methicillin-resistant *S. aureus* displayed elevated (p)ppGpp levels due to a mutation affecting (p)ppGpp hydrolase activity, and other studies have implicated the (p)ppGpp signalling pathway in both early and late stages of human infection, including initial adhesion, invasion of host cells, immune evasion and biofilm formation. As a result, researchers have been working to develop stringent response inhibitors, such as the ppGpp analogue, relacin, and while relacin-mediated inhibition of the stringent response has been reported *in vitro*, no successful clinical interventions have been described to date. Given the ubiquity and clinical significance of the (p)ppGpp-mediated stringent response, it is critical that we continue to develop our understanding of this complex bacterial stress response.

## Abbreviations

ACT: aspartokinase, chorismate mutase, TyrR
EF: elongation factor
HD: hydrolase domain
IF: initiation factor
ppGpp: guanosine tetraphosphate
pppGpp: guanosine pentaphosphate
RA-GTPases: ribosome associated GTPases
RF: release factor
RNAP: RNA polymerase
RRF: ribosome recycling factor
RRM: RNA recognition motif
RSH: RelA SpoT Homologue
SAH: small alarmone hydrolase
SAS: small alarmone synthetase
SD: synthetase domain
ssDNA: single stranded DNA
TGS: Thr-tRNA synthase, GTPase, SpoT
ZFD: zinc finger domain
